# Hydrogen sulfide promotes proliferation and regeneration of human cerebral microvascular endothelial cells via the sonic hedgehog signaling pathway

**DOI:** 10.1515/biol-2025-1242

**Published:** 2026-01-28

**Authors:** Hai-Yan Chen, Jian-Min Huang, Pin Zheng, Gui-Xin Yang, Bing-Bing Qin, Meng-Xue Zang, Jie Wang, Xue-Bin Li

**Affiliations:** The First Clinical Medical College of Jinan University, Guangzhou, 510000, China; Department of Neurology, The Affiliated Hospital of Youjiang Medical University for Nationalities, Baise, 533000, China; Key Laboratory of Research on Clinical Molecular Diagnosis for High Incidence Diseases in Western Guangxi of Guangxi Higher Education Institutions, Baise, 533000, China; Department of Gastroenterology, The Affiliated Hospital of Youjiang Medical University for Nationalities, Baise, 533000, China; Department of Nephrology, The Affiliated Hospital of Youjiang Medical University for Nationalities, Baise, 533000, China; Youjiang Medical University for Nationalities, Baise, 533000, China

**Keywords:** angiogenesis, HCMEC/D3, hypoxia, H_2_S, SHH signaling pathway

## Abstract

We investigated the effects of hydrogen sulfide (H_2_S) and sonic hedgehog (SHH) on the proliferation, autophagy, and apoptosis of human microvascular endothelial cells (HCMEC/D3). We also explored the regulatory relationship between cystathionine-β-synthase (CBS) and the SHH pathway. Human microglia cells (HMC3) were stimulated under hypoxia to secrete H_2_S and SHH proteins, which were then co-cultured with HCMEC/D3 cells. The relationship between H_2_S and SHH was investigated by inhibiting the CBS or SHH pathways. Vascular endothelial growth factor (VEGF) and H_2_S levels were detected using ELISA. The mRNA and Protein levels of VEGF, Beclin-1, light chain 3 (LC3), Cysteine aspartic acid protease-3(caspase-3), CBS, SHH, extracellular regulated kinase 1/2 (ERK1/2) and phospho-ERK1/2 (P-ERK1/2) were determined by RT-PCR and western blot. The results indicated that H_2_S secretion by HMC3 increased during hypoxia, with both CBS and SHH proteins being up-regulated. The inhibition of CBS resulted in decreased levels of H_2_S and SHH in HMC3. When the SHH pathway is inhibited, H_2_S secretion levels remain unaffected. H_2_S and SHH proteins increased VEGF, P-ERK1/2, Beclin-1, and LC3 expression while reducing caspase-3 expression in HCMEC/D3 cells. H_2_S secretion by HMC3 promotes the proliferation and regeneration of HCMEC/D3 by regulating SHH protein and alleviating hypoxic injury.

## 1 Introduction

Cardiovascular and cerebrovascular diseases (CCVDs) comprise a spectrum of pathological conditions that impair the vascular systems of the brain and heart. The incidence of CCVDs has increased in parallel with changes in dietary patterns, and these conditions are now affecting progressively younger populations. Epidemiological studies indicate that 10 %–15 % of stroke cases occur in individuals aged 18–49 years [1]. Among CCVDs, ischemic heart disease and ischemic stroke are the predominant causes of mortality worldwide, contributing to 16 % and 11 % of global deaths, respectively [2].

During ischemic cerebral infarction, cells in the peri-infarct region are exposed to hypoxic stress and may undergo necrosis. A primary therapeutic goal is the restoration of adequate blood flow and nutrient delivery [3], 4]. The formation of collateral circulation is essential for supplying oxygen (O_2_) and nutrients to ischemic tissues and thereby facilitating recovery following stroke [5], 6]. This process depends on the proliferation of vascular endothelial cells and neovascularization, which are regulated by multiple factors, including hemodynamic forces, proteins, cytokines, and endogenous gaseous molecules [7], [8], [9], [10].

Hydrogen sulfide (H_2_S), an endogenous gaseous signaling molecule, is widely distributed in mammalian tissues and exerts critical regulatory effects on the cardiovascular and nervous systems [11], [12], [13]. In mammalian cells, H_2_S is primarily synthesized by the enzymes cystathionine γ-lyase (CSE), cystathionine β-synthase (CBS), 3-mercaptopyruvate sulfurtransferase (3-MST), and cysteine aminotransferase (CAT) [14]. Within the central nervous system (CNS), CBS – predominantly expressed in human microglial cells (HMC3) – catalyzes the production of H_2_S from L-cysteine. Under hypoxic conditions, activated HMC3 cells secrete increased levels of H_2_S [15], 16]. H_2_S has been shown to mitigate secondary injury associated with cerebral ischemia-reperfusion through multiple mechanisms, including attenuation of oxidative stress, suppression of neuroinflammation, inhibition of apoptosis, reduction of cerebrovascular endothelial injury, and modulation of autophagy [17], 18]. It also promotes cellular homeostasis by enhancing phosphorylation of protein kinase B (AKT) and extracellular signal-regulated kinase (ERK), and by upregulating vascular endothelial growth factor (VEGF) and angiopoietin-1 (Ang-1) [14], 19]. While the individual roles of hydrogen sulfide (H_2_S) and sonic hedgehog (SHH) in vascular homeostasis have been investigated, their potential interaction under hypoxic conditions – particularly in the regulation of endothelial cell autophagy and regeneration – remains poorly understood.

The Hedgehog (Hh) signaling pathway, particularly sonic hedgehog (SHH), plays an essential role in regulating cell proliferation, survival, and differentiation. SHH is the most extensively expressed member of the Hh protein family and is critical for the development of both neural and vascular systems. Upon binding to the transmembrane receptor patched-1 (PTCH1), SHH disrupts PTCH1-mediated inhibition of smoothened (SMO), thereby initiating a signaling cascade that activates GLI family transcription factors (GLI1–3) and downstream gene expression. This constitutes the canonical SHH signaling pathway [20]. In addition, two non-canonical SHH pathways, which function independently of GLI-mediated transcription, have been described [21].

SHH signaling is essential not only for organogenesis and vasculogenesis but also for neurovascular repair following hypoxic brain injury. Its activation has been associated with reduced oxidative damage, lipid peroxidation, and apoptosis following focal ischemia-reperfusion [22], [23], [24]. Experimental evidence indicates that exogenous SHH enhances VEGF expression, increases microvascular density, and promotes angiogenesis [25]. Furthermore, the SHH pathway has been implicated in the regulation of autophagy and maintenance of cellular homeostasis, particularly under ischemic conditions [14], [26], [27], [28].

Emerging studies have also suggested that H_2_S may regulate the SHH signalling pathway in cancer models, indicating a broader biological relevance of this interaction [29]. However, it is unclear whether such a regulatory mechanism exists in cerebral endothelial cells under hypoxic conditions. Although it has been proven that H_2_S and SHH can regulate the proliferation of vascular endothelial cells and autophagy, the influence between H_2_S and SHH remains unclear. Therefore, we hypothesized that H_2_S promotes the proliferation and autophagy of vascular endothelial cells injured by hypoxia via the SHH signaling pathway, while concurrently inhibiting apoptosis and alleviating hypoxic injury. Targeting the H_2_S-SHH pathway might contribute to the therapies for ischemic stroke by promoting angiogenesis.

## 2 Materials and methods

### 2.1 Cell culture

HMC3 and human cerebral microvascular endothelial cells (HCMEC/D3) were obtained from Procell (Wuhan, China) and Cellverse (Shanghai, China), respectively. Cells were cultured in Dulbecco’s Modified Eagle Medium (DMEM; 11095080, Gibco, Paisley, Scotland, UK) supplemented with 10 % fetal bovine serum (FBS; WXBD6765V, Sigma, Uruguayan origin, South Africa). Cultures were maintained at 37 °C in a humidified incubator with 5 % carbon dioxide (CO_2_). Antibiotics were not used in any of the experimental procedures.

### 2.2 Co-culture and hypoxia treatment

We used a Transwell insert that allows only paracrine crosstalk between HMC3 microglia (lower well) and HCMEC/D3 endothelial cells (upper insert), a setup previously validated for studying microglial modulation of barrier and angiogenic functions under hypoxia. Sharing the same hypoxic/normoxic milieu, both cell types experience simultaneous oxygen stress while soluble mediators (including H_2_S/SHH) can freely diffuse, enabling us to isolate the microglial paracrine impact on endothelial regeneration. Human brain HCMEC/D3 and human HMC3 were inoculated into the upper and lower compartments of Transwell coculture systems (NEST, Wuxi, China) at a density of 2 × 10^5^/mL. The experimental groups included a control group, a hypoxia group, a hypoxia + cyclopamine group, and a hypoxia + aminooxyacetic acid (AOAA) group. For hypoxic culture conditions, the cell culture plates were placed in an anaerobic tank (Mitsubishi Gas Chemical Company, Inc. Japan), along with an anaerobic production bag, type:C-33,Mitsubishi Gas Chemical Company, Inc. Japan and an oxygen indicator, type:C-22,Mitsubishi Gas Chemical Company, Inc. Japan). it is a gas-based hypoxia model. In hypoxic treatment for 8 h, the indicator is pink, and the oxygen (O_2_) concentration in the anaerobic chamber is less than 0.1 %. Hypoxic treatment for 8 h after reoxygenation, the culture medium was replaced with fresh medium, and cells were cultured for an additional 24 h. The hypoxia + cyclopamine group was cultured with fresh medium containing 10 μM cyclopamine (XW44495181, Macklin) for 24 h. The hypoxia + AOAA group was cultured with fresh medium containing AOAA (5 μM) (XW29211441, Macklin), a CBS inhibitor, forms a covalent oxime bond with CBS, thereby reducing H_2_S production. To put it simply, 8 h hypoxia treatment was performed after co-culture, the medium was replaced after the end of hypoxia, and then routine culture was continued for 24 h to collect media and cells.

Cyclopamine inhibits the SHH signaling pathway by targeting smoothened (SMO) receptors, thereby blocking SHH protein function. AOAA is a CBS inhibitor. AOAA covalently binds to CBS to form external aldehydamine with oxime bond, which inhibits CBS activity and thus inhibits H_2_S secretion.

**Informed consent:** Informed consent has been obtained from all individuals included in this study.

**Ethical approval:** The research related to human use has been complied with all the relevant national regulations, institutional policies and in accordance with the tenets of the Helsinki Declaration, and has been approved by the Medical Ethics Committee of the Affiliated Hospital of Youjiang Medical College for Nationalities. (Ethics Approval No. YYFY-LL-2023-039).

### 2.3 Quantification of H_2_S and VEGF levels

The concentration of H_2_S in the culture supernatant was measured using a colorimetric H_2_S detection kit (ml076943, Mlbio, Shanghai, China) after 24 h of reoxygenation. All procedures were conducted in accordance with the manufacturer’s instructions. The quantification were repeated three times technically and three times biologically. H_2_S concentration (nmol/mL) was calculated using the following formula:

$$H_{2}S\left(\text{nmol}/\text{mL}\right)=\Delta A/0.0022\times V\text{ inverse total}/V\text{ sample}$$

where ΔA: OD, *V* inverse total: total reaction volume, *V* sample: sample volume in reaction).

VEGF levels in the supernatant were quantified using an enzyme-linked immunosorbent assay (ELISA) kit (EK183-96, Multi Sciences, Hangzhou, China) according to the manufacturer’s protocol. Absorbance values were measured using a multimode microplate reader (Victor Nivo, PerkinElmer, USA). The quantification were repeated three times technically and three times biologically.

### 2.4 Angiogenesis assay

The angiogenic potential of HCMEC/D3 cells was evaluated using a tube formation assay with conditioned media derived from HMC3 cultures. Supernatants were collected from HMC3 cells that had been subjected to 8 h of hypoxia followed by a 24-h treatment with or without pharmacological inhibitors under normoxic conditions. These conditioned media were used to resuspend HCMEC/D3 cells in each experimental group.

HCMEC/D3 cells were seeded at a density of 5 × 10^4^ cells per well into 48-well plates pre-coated with 50 μL of Matrigel (C0371, Beyotime, Shanghai, China) per well. After 4.5 h of incubation, angiogenic activity was assessed by quantifying the formation of capillary-like structures. Tube formation was analyzed in three randomly selected microscopic fields per well at 10 × magnification using an inverted phase-contrast microscope. Use ImageJ software for calculation and evaluate using master segments lens, meshes, toll lens, and branching lens. The assay were repeated three times technically and three times biologically.

### 2.5 Reverse transcription-polymerase chain reaction (RT-PCR)

HMC3 and HCMEC/D3 cells were harvested after 24 h of reoxygenation for RT-PCR analysis. TRNAiso Plus (9109, Takara, Beijing, China) was used to extract RNA from both cell types, and we measured RNA concentrations spectrophotometrically. Complementary DNA (cDNA) was synthesized from 500 ng of total RNA using a reverse transcription kit (RR047A, Takara, Beijing, China), following the manufacturer’s protocol.

Gene expression levels of *VEGF*, hypoxia-inducible factor-1 alpha (*HIF-1α*), caspase-3 (*CASP3*), Beclin-1 (*BECN1*), microtubule-associated protein light chain 3 (*MAP1LC3*), and the reference gene glyceraldehyde-3-phosphate dehydrogenase (*GAPDH*) were assessed using a LightCycler^®^ 96 system (Roche, Basel, Switzerland).

Each PCR reaction was carried out in a total volume of 20 μL, consisting of 10 μL TB Green^®^ Premix Ex Taq™ II (RR820A, Tli RNaseH Plus; Takara), 2 μL cDNA template, 1 μL of gene-specific primers (Table 1), and 6 μL nuclease-free water. The thermal cycling conditions were as follows: initial denaturation at 95 °C for 30 s, followed by 40 cycles of denaturation at 95 °C for 8 s and combined annealing/extension at 60 °C for 30 s.

**Table 1:** Primer sequences used in RT-PCR.

mRNA	Forward (5′–3′)	Reverse (5′–3′)
*BCL1*	CCA​TGC​AGG​TGA​GCT​TCG​T	GAA​TCT​GCG​AGA​GAC​ACC​ATC
*LC3*	AAC​ATG​AGC​GAG​TTG​GTC​AAG	GCT​CGT​AGA​TGT​CCG​CGA​T
*VEGF*	GCG​CTC​GGT​GCT​GGA​ATT​TG	TAG​AGC​AAT​CTC​CCC​AAG​CCG​TCG
*CASP3*	TGG​TAC​AGA​TGT​CGA​TGC​AGC	AGG​TCA​ACA​GGT​CCA​TTT​GTT​C
*HIF1A*	TTC​ACC​TGA​GCC​TAA​TAG​TCC	CAA​GTC​TAA​ATC​TGT​GTC​CTG
*GAPDH*	TGA​CAT​CAA​GAA​GGT​GGT​GAA​GCA​G	GTG​TCG​CTG​TTG​AAG​TCA​GAG​GAG

Amplification specificity was confirmed by melting curve analysis, which showed single peaks for each target gene, indicating the absence of non-specific amplification. The assay were repeated three times technically and three times biologically. Relative mRNA expression levels were calculated using the 2^−ΔΔCt^ method. For each sample, the cycle threshold (Ct) value of *GAPDH* was subtracted from the Ct value of the target gene to obtain ΔCt, and differences between experimental and control groups were expressed as ΔΔCt.

### 2.6 Western blot analysis

Total protein was extracted from HMC3 and HCMEC/D3 cells using RIPA lysis buffer (P0013B, Beyotime, Shanghai, China), and protein concentrations were determined using the BCA Protein Assay Kit (P0012, Beyotime, Shanghai, China) following the manufacturer’s instructions. Equal amounts of protein (20 μg/lane) were separated by sodium dodecyl sulfate-polyacrylamide gel electrophoresis (SDS-PAGE) and transferred onto hydrophobic polyvinylidene fluoride (PVDF) membranes (Immobilon-P, Millipore Corporation, Billerica, MA, USA). The assay were repeated three times technically and three times biologically. Membranes were blocked with protein-free rapid blocking buffer (P0013B, Yamei, Shanghai, China) for 2 h at room temperature to prevent nonspecific binding. Following blocking, membranes were washed five times for 5 min each with Tris-buffered saline containing 0.1 % Tween 20 (TBS-T) and incubated overnight at 4 °C with primary antibodies.

The following primary antibodies were used (all from Proteintech Group, Wuhan, China unless otherwise noted): mouse anti-caspase-3/p17/p19 monoclonal antibody (66470-2-Ig), mouse anti-Beclin-1 monoclonal antibody (66665-1-Ig), rabbit anti-LC3 polyclonal antibody (14600-1-AP), rabbit anti-SHH polyclonal antibody (20697-1-AP), mouse anti-CBS monoclonal antibody (67861-1-Ig), rabbit anti-ERK1/2 polyclonal antibody (11257-1-AP), and rabbit anti-phospho-ERK1/2 (Thr202/Tyr204) polyclonal antibody (28733-1-AP). Rabbit anti-GAPDH polyclonal antibody (bs-2188R, Bioss, Beijing, China) was used as a loading control. All primary antibodies were diluted 1:1,000, except for anti-GAPDH, which was diluted 1:2000. Dilutions were prepared in TBS-T supplemented with 5 % non-fat milk. We used Ponceau S prior to antibody probing. After primary antibody incubation, membranes were washed five times for 5 min each with TBS-T and subsequently incubated with horseradish peroxidase (HRP)-conjugated secondary antibodies – either anti-rabbit immunoglobulinG (IgG) (A0208) or anti-mouse IgG (SA00001-1) (Proteintech Group, Wuhan, China) – diluted 1:20,000 in TBS-T containing 5 % non-fat milk.

Final washes were performed (five times for 5 min each) using TBS-T. Immunoreactive bands were visualized using enhanced chemiluminescence (ECL) with the Omni-ECL™ ultra-sensitive detection kit (SQ201, YaMei, Shanghai, China), and images were captured using a gel imaging system (Thermo Fisher Scientific, Waltham, MA, USA). Densitometric analysis was performed using ImageJ software (National Institutes of Health, Bethesda, MD, USA). Protein expression levels were normalized to GAPDH as an internal control,WB were repeated three times technically and three times biologically.

### 2.7 Data and statistical analyses

All statistical analyses were performed using GraphPad Prism version 9.0 (GraphPad Software, San Diego, CA, USA). For datasets obtained from RT-PCR, western blotting, H_2_S quantification, and ELISA assays, the Kolmogorov–Smirnov test was applied to evaluate the normality of data distribution.

Data conforming to a normal distribution are presented as mean ± standard deviation (SD). Comparisons among multiple groups were conducted using one-way analysis of variance (ANOVA), followed by post hoc multiple comparisons testing as appropriate. A *p*-value of less than 0.05 was considered indicative of statistical significance.

## 3 Results

### 3.1 Hypoxia stimulates H_2_S secretion by HMC3 cells

**Figure 1:**
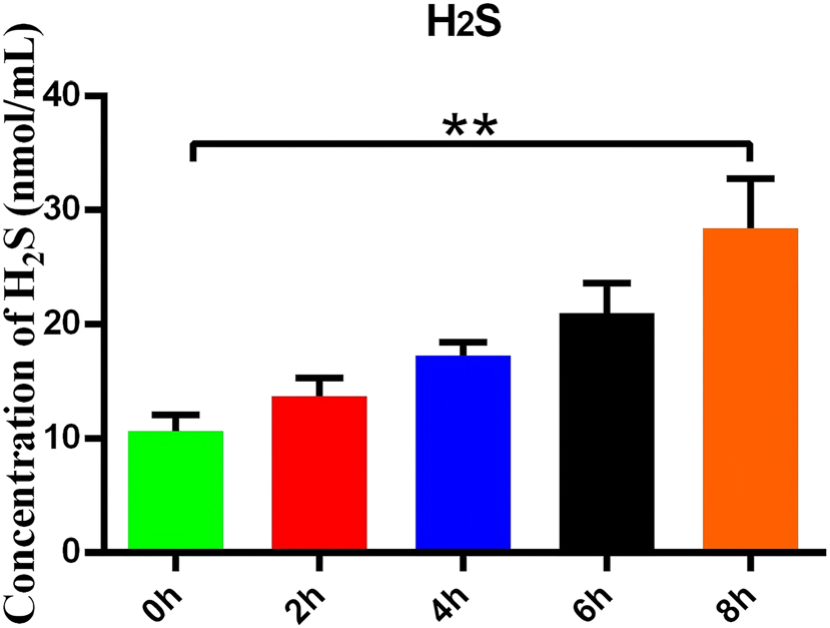
Hypoxia stimulates H_2_S secretion by HMC3 cells. H_2_S concentrations in the culture medium were measured following exposure of HMC3 cells to varying durations of hypoxia. Data are represented as mean ± SD **p < 0.01, compared with the 0-h group.

To investigate the stimulatory effect of hypoxia on H_2_S secretion by HMC3 cells, we treat HMC3 cells with varying durations of hypoxic conditions and subsequently measured the H_2_S concentration in the culture medium. The results demonstrated a significant increase in H_2_S content in the medium after 8 h of hypoxia compared to baseline (*P* < 0.01) (Figure 1).

### 3.2 Hypoxia-induced *HIF1A* activation correlates with enhanced H_2_S secretion

**Figure 2:**
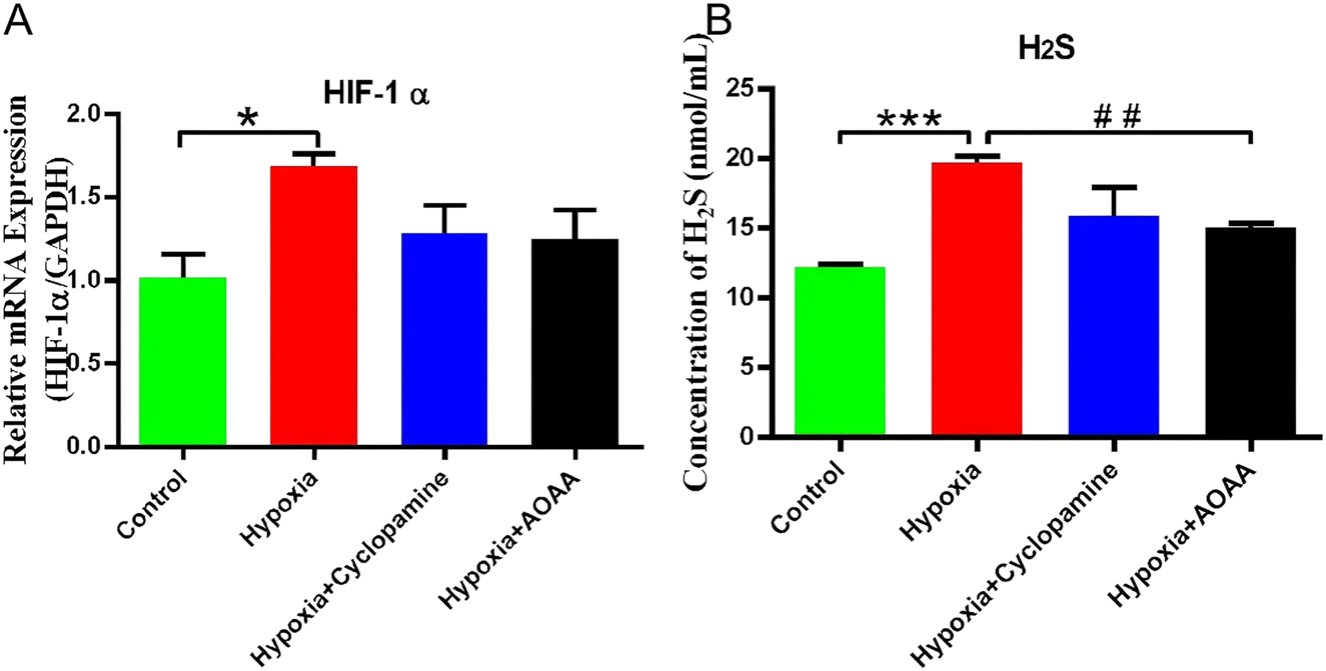
Effects of hypoxic stimulation on HMC3 cells. (A) *HIF1A* mRNA expression levels in HMC3 cells under normoxic and hypoxic conditions. (B) H_2_S concentration in the culture medium. Data are represented as mean ± SD *p < 0.05, ***p < 0.005, compared with the control group; ^##^ *p* < 0.01, compared with the hypoxia group.

Our findings revealed that hypoxia significantly upregulated HIF-1α mRNA expression in HMC3 cells compared to the control group (*P* < 0.05). However, HIF-1α mRNA levels did not differ significantly between the hypoxia group and the hypoxia + cyclopamine group (*P* > 0.05). Meanwhile, there was no significant difference in the expression of HIF-1α mRNA between hypoxia + AOAA group and hypoxia group (*P* > 0.05) (Figure 2A).

Measurement of H_2_S content revealed that hypoxia significantly increased H_2_S secretion compared to the control group (*p* < 0.005). The H_2_S content in the hypoxia + cyclopamine group was slightly decreased, but there was no significant change in H_2_S content between the hypoxia + cyclopamine group and the hypoxia alone group (*P* > 0.05). In contrast, treatment with AOAA under hypoxic conditions resulted in a significant decrease in H_2_S concentration compared to the hypoxia group (*p* < 0.01) (Figure 2B).

Collectively, these findings indicate that hypoxia induces *HIF1A* transcriptional activation in HMC3 cells and enhances H_2_S secretion. Inhibition of CBS via AOAA significantly attenuates this hypoxia-induced increase in H_2_S, suggesting a regulatory role of CBS in mediating H_2_S production under hypoxic conditions. Additionally, it is worth noting that although HIF-1 *α* mRNA did not reach statistical significance, a significant downward trend was observed after treatment with cyclic amine and AOAA.

Although AOAA produced the only statistically significant decrease in H_2_S and cyclopropylamine also reduced the mean, indicating that this is a moderate but consistent effect, not solely driven by statistical thresholds.

### 3.3 H_2_S levels regulate SHH protein expression

To further clarify the role of CBS in mediating H_2_S synthesis and its potential regulatory effect on the SHH signaling pathway, HMC3 cells were subjected to hypoxia with or without pharmacological inhibition of CBS or SHH. Protein expression levels of CBS and SHH were subsequently analyzed.

Compared with the control group, CBS protein expression was not significantly altered in either the control + cyclopamine or control + AOAA groups (*p* > 0.05). However, hypoxia significantly increased CBS protein levels relative to the control group (*p* < 0.01). No significant difference in CBS expression was observed between the hypoxia and hypoxia + cyclopamine groups (*p* > 0.05), although the comparison between hypoxia and hypoxia + cyclopamine did not achieve statistical significance, CBS band intensity exhibited a consistent downward shift after cyclopamine exposure, whereas a significant reduction in CBS protein levels was detected in the hypoxia + AOAA group compared to the hypoxia group (*p* < 0.01).

**Figure 3:**
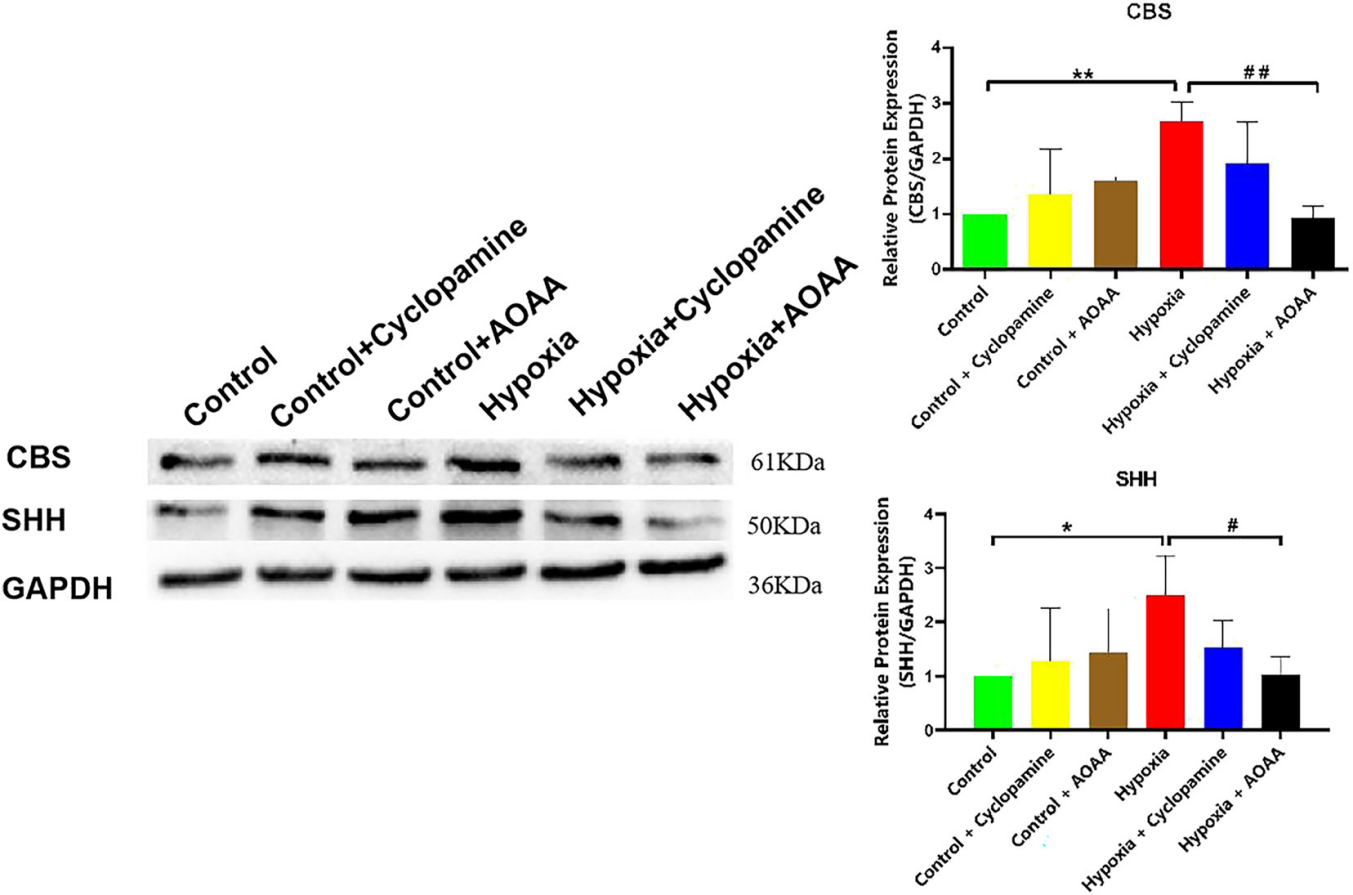
Effects of CBS and SHH pathway inhibition on CBS and SHH protein expression. Data are represented as mean ± SD *p < 0.05, compared with the control group; ^#^ *p* < 0.05, compared with the hypoxia group.

Similarly, SHH protein expression remained unchanged in the control + cyclopamine and control + AOAA groups relative to the control group (*p* > 0.05), but was significantly elevated in the hypoxia group (*p* < 0.05). There was no significant change in the SHH protein level in the hypoxia + cyclopamine group (*P* > 0.05); however, SHH levels were significantly decreased in the hypoxia + AOAA group compared to the hypoxia group (*p* < 0.05) (Figure 3).

These findings indicate that CBS upregulation under hypoxic conditions enhances both H_2_S and SHH protein expression in HMC3 cells. Inhibition of CBS resulted in reduced expression of both CBS and SHH proteins, suggesting that CBS regulates SHH synthesis through modulation of H_2_S levels. Conversely, inhibition of the SHH signaling pathway did not alter CBS expression, indicating that SHH does not regulate CBS protein synthesis under these conditions, and H_2_S upregulates SHH under hypoxia via CBS-dependent pathways. Moreover, Although the SHH signal in the hypoxia + cyclopamine group did not reach statistical significance, its band intensity consistently decreased relative to the hypoxia control, implying an inhibitory tendency that should be acknowledged alongside the significant AOAA-induced reduction.

### 3.4 H_2_S promotes tube formation in HCMEC/D3 via the SHH signaling pathway

**Figure 4:**
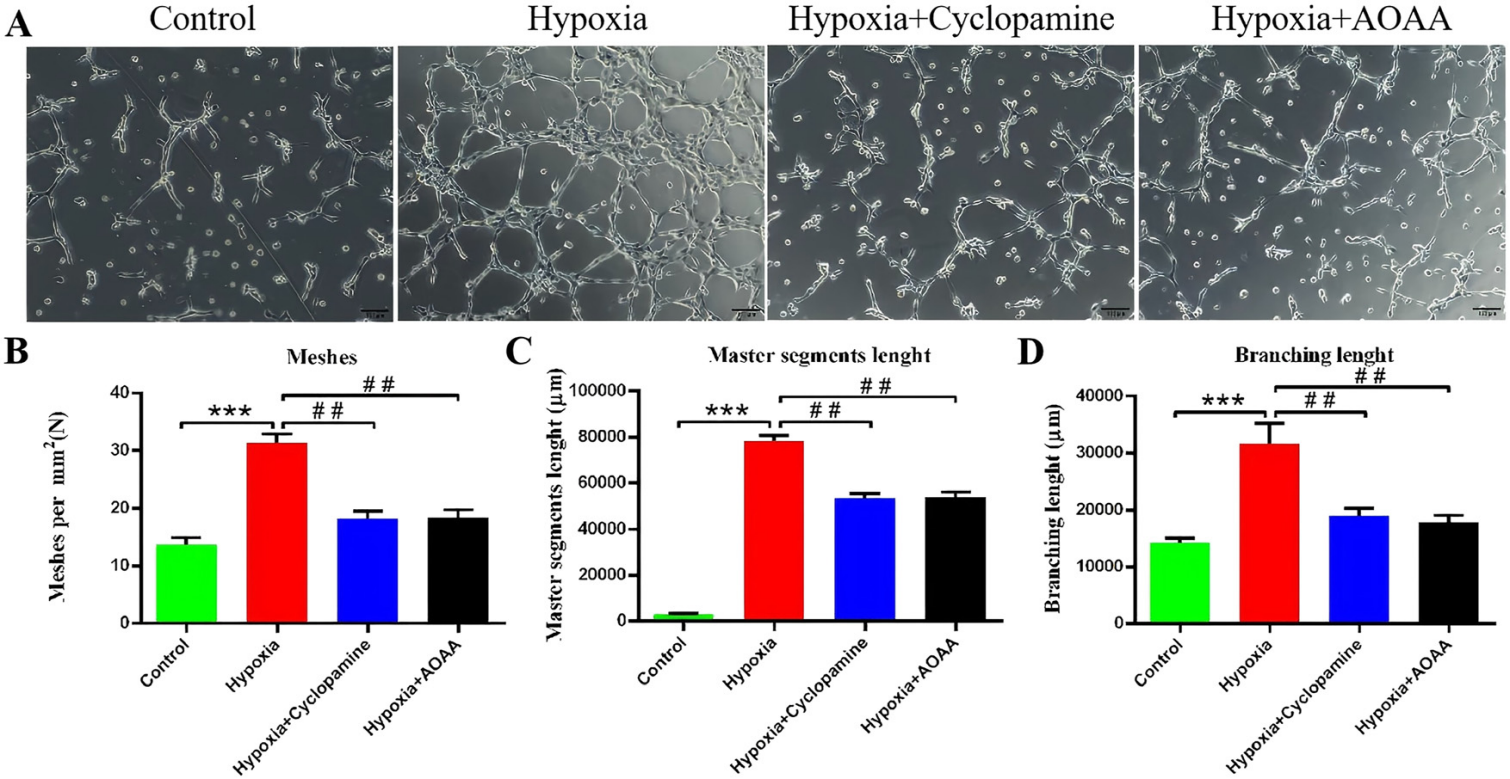
Effects of H_2_S and SHH signaling pathways on angiogenesis in HCMEC/D3 cells. (A) Representative images of tube formation by HCMEC/D3 cells after 4.5 h of incubation under different treatment conditions. (B) Quantification of mesh number per mm^2^. (C) Quantification of main trunk length. (D) quantification of branch length. Data are presented as mean ± SD ***p < 0.005 versus control group; ^##^ *p* < 0.01 versus hypoxia group.teaser-image

To assess the effects of H_2_S and the SHH signaling pathway on angiogenic activity in HCMEC/D3 cells, tube formation assays were performed using conditioned media from HMC3 cultures subjected to various experimental conditions (Figure 4A).

The results of mesh formation numbers showed that compared with the control group, the number of mesh per mm^2^ in hypoxia group was significantly increased (*P* < 0.005). Compared to the hypoxia group, the number of meshes per mm^2^ in the hypoxia + AOAA group was significantly reduced. (*P* < 0.01) (Figure 4B).

Similarly, the main trunk length of the tubular structures was significantly increased in the hypoxia group compared to the control (*p* < 0.005). Both cyclopamine and AOAA treatments under hypoxic conditions resulted in a significant reduction in main trunk length compared to the hypoxia group (*p* < 0.01) (Figure 4C).

Branch length analysis also showed a significant increase in the hypoxia group relative to the control group (*p* < 0.005). Treatment with either cyclopamine or AOAA significantly decreased branch length when compared to the hypoxia group (*p* < 0.01 for both) (Figure 4D).

Collectively, these results suggest that H_2_S promotes endothelial cell proliferation and migration, facilitating neovascularization in HCMEC/D3 cells via activation of the SHH signaling pathway.

### 3.5 H_2_S-induced SHH signaling promotes VEGF secretion and ERK activation in HCMEC/D3 cells

Building upon earlier findings demonstrating hypoxia-induced secretion of H_2_S and SHH proteins in HMC3 cells, a co-culture model was employed to examine the downstream effects of these proteins on HCMEC/D3 cells. Specifically, the expression of VEGF and ERK signaling components was evaluated to investigate the mechanism underlying H_2_S- and SHH-mediated endothelial proliferation.

**Figure 5:**
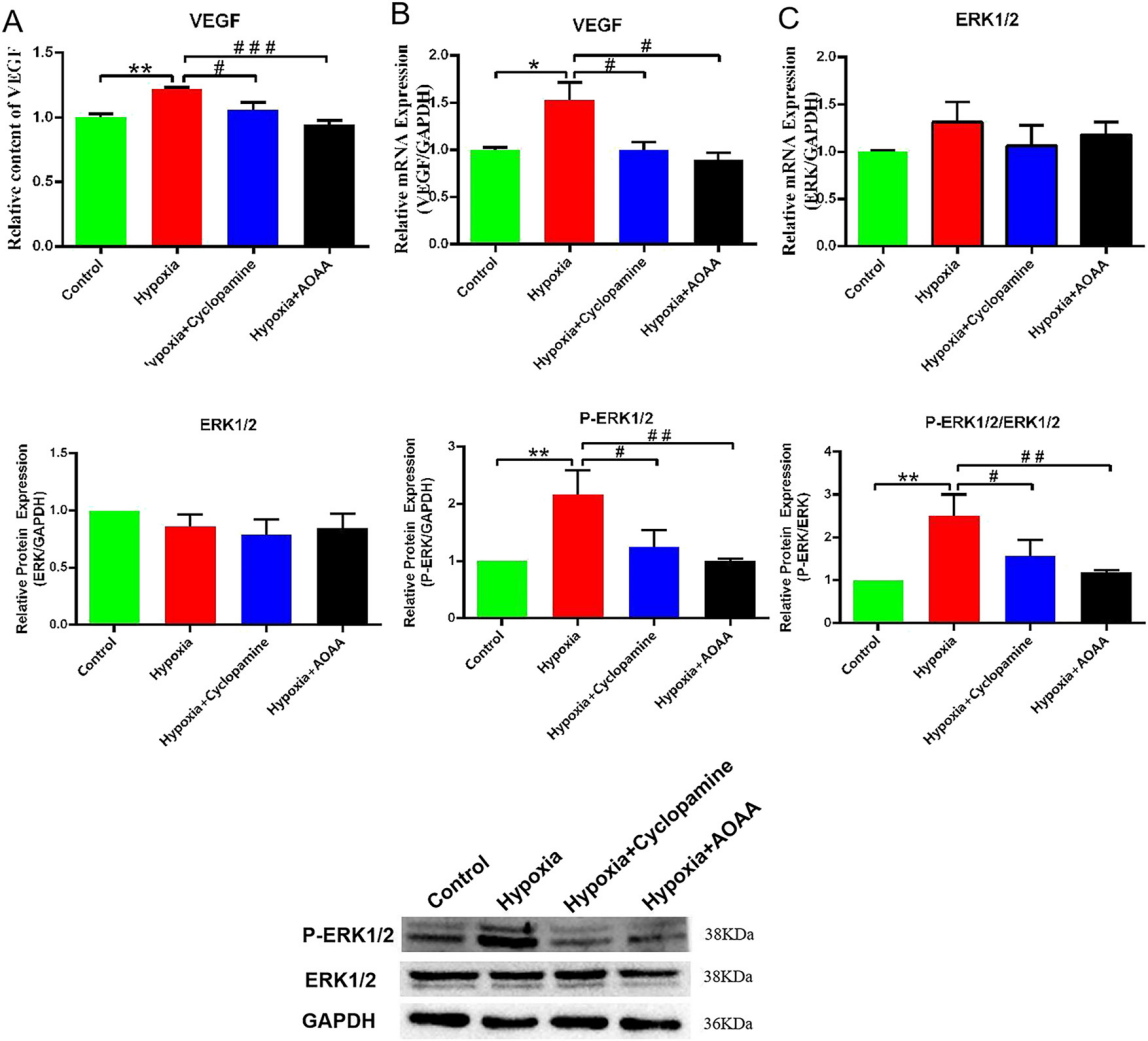
VEGF expression and ERK1/2 phosphorylation in HCMEC/D3 cells. (A) VEGF protein levels in the culture medium. (B) Relative *VEGF* mRNA expression levels. (C) Western blot analysis of total ERK1/2 and phosphorylated ERK1/2 (P-ERK1/2) protein levels. Data are presented as mean ± SD*p < 0.05, **p < 0.01, compared with the control group; ^#^ *p* < 0.05, ^##^ *p* < 0.01, ^###^ *p* < 0.005, compared with the hypoxia group.

VEGF protein levels in the culture medium were significantly elevated in the hypoxia group compared to the control group (*p* < 0.01). In contrast, both the hypoxia + cyclopamine and hypoxia + AOAA groups exhibited significantly reduced VEGF concentrations relative to the hypoxia group (*p* < 0.05 and *p* < 0.005, respectively) (Figure 5A). Similarly, *VEGF* mRNA expression was significantly upregulated in the hypoxia group compared to control (*p* < 0.05). This increase was significantly attenuated in both the hypoxia + cyclopamine and hypoxia + AOAA groups relative to the hypoxia group (*p* < 0.05 for both) (Figure 5B).

Western blot analysis revealed no significant difference in total ERK1/2 protein expression across the experimental groups (*p* > 0.05). However, the ratio of P-ERK1/2 to total ERK1/2 was significantly elevated in the hypoxia group compared to the control group (*p* < 0.01). This ratio was significantly reduced in the hypoxia + cyclopamine group (*p* < 0.05) and further decreased in the hypoxia + AOAA group (*p* < 0.01) compared to the hypoxia group (Figure 5C).

These results suggest that under hypoxic conditions, H_2_S promotes VEGF secretion and ERK1/2 phosphorylation in HCMEC/D3 cells via activation of the SHH signaling pathway. This mechanism likely contributes to enhanced endothelial proliferation and angiogenesis.

### 3.6 H_2_S promotes autophagy and alleviates apoptosis via SHH signaling pathway

To determine whether the pro-survival effect of microglial H_2_S/SHH on endothelial cells is linked to changes in cell-death and self-renewal pathways, we next examined autophagy and apoptosis. Focusing on these two processes allows us to clarify how the H_2_S–SHH axis tips the balance between endothelial preservation and injury under hypoxic stress. Therefore,we evaluated the effects of H_2_S and SHH signaling on autophagy and apoptosis in hypoxia-injured HCMEC/D3 cells, the expression of relevant autophagy- and apoptosis-associated genes and proteins was assessed in co-cultured HCMEC/D3 cells.

In this study, RT-PCR analysis showed that *BECN1* mRNA levels were significantly elevated in the hypoxia group compared to the control group (*p* < 0.05). However, *BECN1* mRNA levels were significantly decreased in both the hypoxia + cyclopamine and hypoxia + AOAA groups compared to the hypoxia group (*p* < 0.01 for both). A similar expression pattern was observed for *MAP1LC3* mRNA.

**Figure 6:**
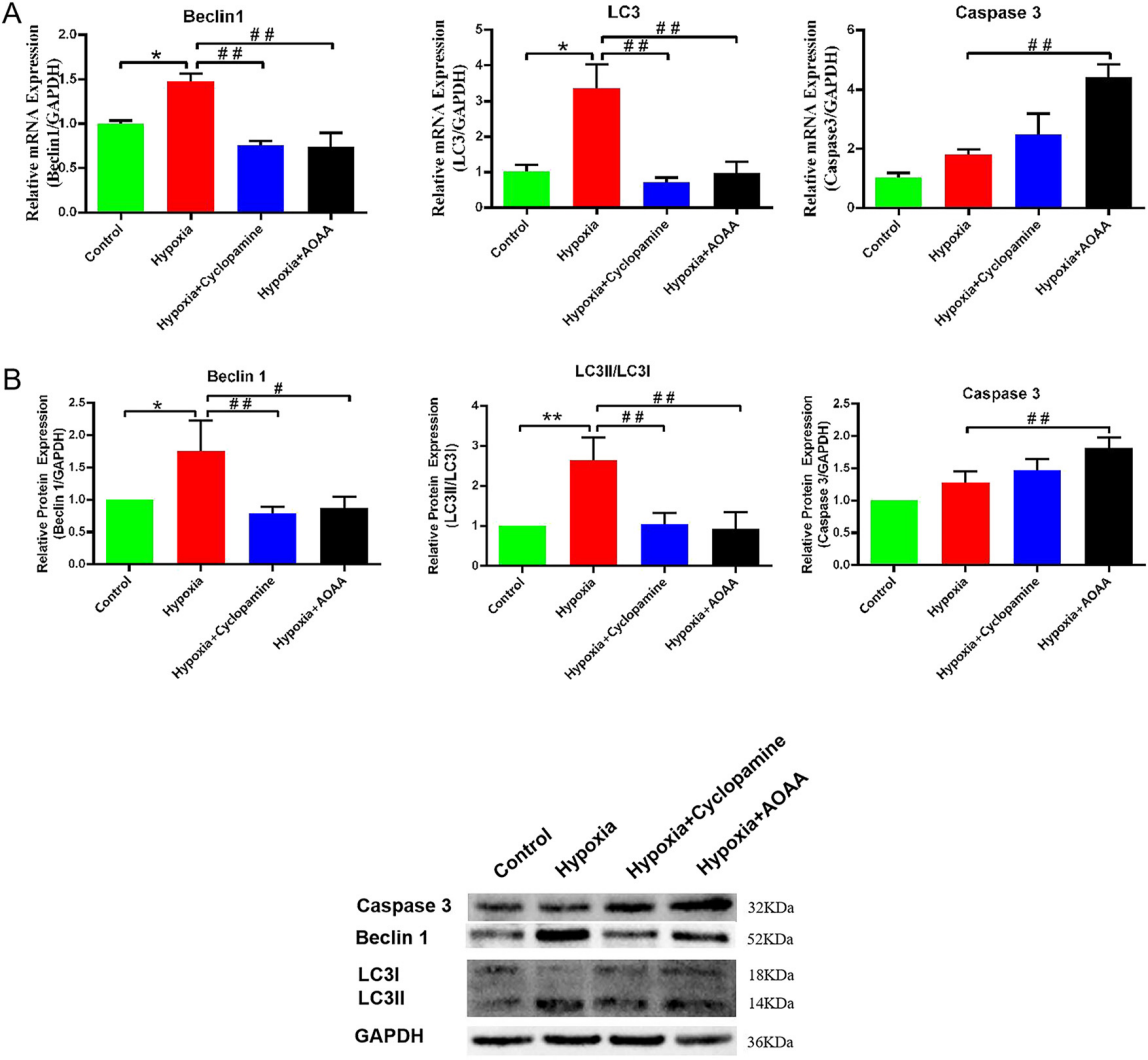
Effects of H_2_S and SHH signaling on autophagy and apoptosis in HCMEC/D3 cells. (A) Relative mRNA expression levels of *CASP3*, *BCL1*, and *LC3* in HCMEC/D3 cells. (B) Protein expression levels of caspase-3, Beclin-1, and LC3 as determined by western blot analysis. Data are presented as mean ± SD *p < 0.05, **p < 0.01, compared with the control group; ^#^ *p* < 0.05, ^##^ *p* < 0.01, compared with the hypoxia group.

*CASP3* mRNA expression did not differ significantly between the hypoxia and control groups (*p* > 0.05). However, compared to the hypoxia group, *CASP3* transcription was significantly upregulated in both the hypoxia + cyclopamine (*p* < 0.05) and hypoxia + AOAA (*p* < 0.01) groups (Figure 6A).

Western blot results corroborated these findings. Beclin-1 protein levels were significantly elevated in the hypoxia group relative to the control (*p* < 0.05), but were significantly reduced in the hypoxia + cyclopamine (*p* < 0.01) and hypoxia + AOAA (*p* < 0.05) groups (Figure 6B). Similarly, the LC3II/LC3I ratio was markedly increased in the hypoxia group compared to control (*p* < 0.01), and significantly decreased in both the hypoxia + cyclopamine and hypoxia + AOAA groups (*p* < 0.01 for both), suggesting reduced autophagic activity with pathway inhibition.

Protein levels of caspase-3 did not differ significantly between the control and hypoxia groups (*p* > 0.05). However, a significant increase in caspase-3 protein expression was observed in the hypoxia + AOAA group compared to the hypoxia group (*p* < 0.01) (Figure 6B).

Collectively, these findings indicate that H_2_S secreted by HMC3 cells under hypoxic conditions promotes autophagy in HCMEC/D3 cells through activation of the SHH signaling pathway, contributing to the removal of damaged cellular components and mitigating hypoxic injury. Concurrently, this pathway appears to attenuate apoptotic responses, thereby reducing cell death and preserving endothelial integrity during hypoxic stress.

## 4 Discussion

In this study, we demonstrate that H_2_S, produced by hypoxia-activated microglia, promotes angiogenesis in HCMEC/D3 cells by activating the SHH signalling pathway. Additionally, H_2_S and SHH modulate autophagy and apoptosis, mitigating hypoxic injury. Ischemic stroke continues to pose a major threat to both quality of life and survival. Restoration of blood flow following ischemic injury largely depends on the formation of collateral circulation, a process intricately linked to the proliferation and regeneration of cerebral microvascular endothelial cells, such as HCMEC/D3. Numerous factors have been identified as key regulators of angiogenesis, including angiogenic proteins (e.g., VEGF, basic fibroblast growth factor), gaseous transmitters (e.g., H_2_S, carbon monoxide (CO)), and intracellular signaling pathways such as SHH, mechanistic target of rapamycin (mTOR), and mitogen-activated protein kinases (MAPK) [30], [31], [32].

Among the enzymes responsible for endogenous H_2_S production, CBS is the predominant isoform in brain tissue, primarily expressed in astrocytes, neurons, and microglia. While astrocytes and neurons mainly facilitate neural signal transmission, microglia act as immune cells that respond rapidly to hypoxic stress, migrating, proliferating, and contributing to repair. Under hypoxic conditions, activated microglia increase CBS expression, leading to enhanced H_2_S production, which supports neurovascular recovery [33], [34], [35]. In this study, HMC3 microglial cells were thus used as the cellular source of H_2_S.

Previous studies have demonstrated that H_2_S upregulates *HIF1A* and *VEGF* at both mRNA and protein levels in brain endothelial cells, enhancing HIF-1α binding activity and promoting angiogenesis under hypoxia [14]. In ischemic models, H_2_S promotes neuroprotection by stimulating AKT and ERK phosphorylation, upregulating VEGF and Ang-1 expression, and enhancing vascular endothelial cell proliferation and migration, thereby facilitating angiogenesis, improving cerebral blood flow, and offering post-ischemic neuroprotection [35], 36]. Likewise, the SHH signaling pathway plays an essential role in angiogenesis and tissue regeneration in adult tissues. SHH indirectly promotes angiogenesis by upregulating VEGF, Ang-1, and Ang-2 expression [37], and enhances ERK1/2 phosphorylation, thereby facilitating endothelial proliferation and vascular remodeling [38].

Upregulation of VEGF expression and ERK1/2 phosphorylation is essential for promoting vascular endothelial cell proliferation and neovascularization. In this context, both the H_2_S and SHH signaling pathways serve as key regulatory mechanisms. The findings of the present study are consistent with these observations, further substantiating the role of H_2_S in promoting angiogenesis. Specifically, our results show that elevated H_2_S levels are associated with increased VEGF expression and ERK1/2 phosphorylation, leading to enhanced endothelial proliferation and tube formation. Unlike VEGF or basic fibroblast growth factor (bFGF), H_2_S uniquely coordinates both ERK phosphorylation and VEGF/Ang-1 expression, and exhibits a more sustained pro-proliferative effect on endothelial cells, highlighting its distinct angiogenic regulatory profile. Conversely, inhibition of either H_2_S synthesis or SHH signaling significantly reduces VEGF and ERK1/2 levels, thereby impairing endothelial cell proliferation. Collectively, this study provides the first direct evidence that H_2_S regulates the SHH signaling pathway to promote angiogenesis under hypoxic conditions in HCMEC/D3 cells.

In addition to angiogenesis, our findings suggest that the H_2_S-SHH axis affects cellular stress responses, particularly autophagy and apoptosis. These processes are crucial for mitigating damage caused by hypoxia. In cardiovascular disease, H_2_S has been shown to attenuate atherosclerosis and reduce plaque vulnerability by promoting autophagy in vascular smooth muscle cells (VSMC), enhancing collagen synthesis, and inhibiting apoptosis [14]. Additional studies have demonstrated that H_2_S can mitigate cellular injury by simultaneously suppressing apoptosis and inducing autophagy [39]. Furthermore, low concentrations of H_2_S have been reported to protect brain tissue following cerebral ischemia–reperfusion injury through modulation of both autophagic and apoptotic pathways [40].

Similarly, the SHH signaling pathway contributes to cellular homeostasis under hypoxic conditions by promoting autophagosome formation in damaged cells [41], [42], [43]. Inhibition of SHH has been linked to enhanced apoptosis in cancer models, and SHH signaling has been shown to regulate apoptosis both *in vitro* and *in vivo* [44], [45], [46]. Recent research shows that activating the Sonic hedgehog (Shh) signaling pathway after middle cerebral artery occlusion/reperfusion injury has multiple effects: enhancing fibrotic scar formation, preserving synaptic structures and promoting new synapse generation, alleviating neurological deficits, reducing cell apoptosis, driving meningeal fibroblast differentiation into myofibroblasts, and facilitating meningeal fibroblast proliferation and migration. These results imply that targeting the Shh signaling pathway could be a new stroke treatment strategy [47]. These findings align with the results of the present study, wherein elevated levels of H_2_S and SHH in hypoxia-damaged vascular endothelial cells enhanced autophagy and suppressed apoptosis. In contrast, inhibition of either H_2_S synthesis or SHH signaling produced the opposite effects.

While previous research has suggested a functional interaction between H_2_S and SHH, direct evidence of H_2_S promoting angiogenesis via regulation of the SHH pathway has been lacking. In this study, we investigated the role of H_2_S in modulating endothelial cell proliferation, autophagy, and apoptosis through SHH signaling under hypoxic conditions. Initially, H_2_S concentrations in the HMC3 culture medium were measured following exposure to different durations of hypoxia. Subsequently, the effects of H_2_S and SHH secreted by HMC3 cells on co-cultured HCMEC/D3 cells were assessed. The results demonstrated that H_2_S regulates SHH protein expression and modulates VEGF levels via the SHH pathway, thereby promoting the proliferation and regeneration of HCMEC/D3 cells. In addition, both H_2_S and SHH contributed to increased ERK phosphorylation, supporting endothelial proliferation. Importantly, H_2_S and SHH alleviated hypoxic injury by enhancing autophagy and inhibiting apoptosis. Overall, our findings suggest that H_2_S regulates hypoxia-induced cell regeneration and homeostasis in HCMEC/D3 cells by modulating the SHH signaling pathway.

The regulatory relationship between H_2_S and the SHH signaling pathway remains incompletely understood. Based on current evidence, it is hypothesized that H_2_S may exert regulatory effects on SHH signaling by modifying key pathway components – such as SMO or GLI transcription factors – via persulfidation, a post-translational modification that can alter protein function and downstream signal transduction [47].

Emerging evidence suggests that H_2_S fine-tunes the SHH cascade through multifaceted redox chemistry: persulfidation of conserved cysteines within the sterol-sensing domain of SMO (e.g. Cys469) can promote cholesterol-dependent ciliary translocation and downstream signal initiation, whereas similar modification of zinc-finger or repressor cysteines in GLI1/2 (e.g. Cys501) may impede ubiquitination or foster dimerization, thereby favoring transcriptional activation; parallels drawn from Wnt and Notch pathways – where disulfide formation in LRP6 or S-nitrosation of NICD modulates signaling – lend further credence to such redox-based regulation, and indirect crosstalk also exists, since H_2_S-derived polysulfides activate Nrf2 via Keap1 persulfidation and activated Nrf2 can occupy the GLI1 promoter, providing an additional transcriptional conduit through which H_2_S feeds into SHH-mediated endothelial responses.

Additionally, H_2_S may indirectly influence SHH pathway activity by modulating oxidative stress and glutathione metabolism [48]. These mechanisms suggest that H_2_S and SHH signaling may interact through both post-translational and redox-dependent regulatory processes. However, the precise molecular pathways underlying this interaction require further experimental validation.

Moreover, the expression of HIF-1α is primarily regulated by oxygen availability and is well established as a canonical response to hypoxia. In the present study, the regulatory interaction between H_2_S and In addition to angiogenesis, our findings suggest that the Hwas specifically evaluated under hypoxic conditions to reflect the pathophysiological environment of ischemic injury and associated angiogenic responses. The finding that *HIF1A* expression remained unaffected by CBS or SHH inhibition supports the conclusion that hypoxia is the dominant regulator of HIF-1α in this context. This observation reinforces the idea that while H_2_S and SHH signaling contribute to downstream cellular responses under hypoxia, they do not directly modulate HIF-1α expression.

In this study, the relationship between CBS inhibition and SHH pathway inhibition was examined to evaluate potential regulatory interactions. Inhibition of CBS led to a reduction in both H_2_S production and SHH protein expression. CBS blockade simultaneously erased hypoxia-induced CBS protein, H_2_S output and SHH, implying that H_2_S persulfidates CBS itself or its transcriptional drivers to establish a positive feedback loop; in contrast, Smo inhibition left CBS intact, confirming a unidirectional CBS-H_2_S → SHH relay. We posit that H_2_S sulfhydrates conserved cysteines within SMO and GLI, amplifying ciliary translocation and DNA binding, thereby up-regulating VEGF/ERK signaling, accelerating autophagic flux and dampening caspase-3 activity in HCMEC/D3. Exploiting this circuitry by H_2_S donors or selective SHH activators could therapeutically reproduce the pro-angiogenic and cytoprotective phenotype required for post-stroke neurovascular repair. In contrast, inhibition of the SHH pathway did not significantly alter CBS protein levels,and we propose that inhibiting SHH does not affect H_2_S secretion. It may be due to the functional independence of the H_2_S synthesis pathway. These findings suggest that H_2_S synthesized by CBS modulates SHH protein expression and influences the functional activity of the SHH signaling pathway. Overall, H_2_S was shown to promote the proliferation and regeneration of HCMEC/D3 cells by regulating SHH signaling and mitigating hypoxia-induced cellular injury.

Angiogenesis following ischemic stroke is critical for restoring cerebral blood flow and promoting neurological recovery. In this study, we demonstrate that H_2_S promotes vascular endothelial cell proliferation, migration, and angiogenesis via activation of the SHH signaling pathway, thereby alleviating hypoxia-induced injury. These findings point to the therapeutic potential of targeting the H_2_S–SHH axis in ischemic stroke and suggest it may represent a promising avenue for future clinical intervention. Future therapeutic strategies could involve pharmacological modulation of H_2_S levels or targeted activation of the SHH pathway to enhance neurovascular repair in stroke patients.

However, this study has several limitations. The *in vitro* co-culture model using HCMEC/D3 cells and HMC3 microglia does not fully replicate the complexity of the *in vivo* environment during cerebral infarction; it lacks blood flow, systemic immune interactions, and the intricate inflammatory milieu and hemodynamic cues present in post-stroke brain tissue. Additionally, potential off-target effects or confounding factors associated with pharmacological inhibition of CBS and SHH cannot be excluded. To address these deficiencies, future studies should validate the findings in transgenic animal models (e.g., endothelial-specific Cbs-knockout mice) and analyze plasma and perilesional tissue from ischemic stroke patients, thereby confirming whether the H_2_S–SHH axis operates similarly *in vivo* and strengthening its translational relevance.
